# The Impact of COVID-19 on the Psychological Well-Being of Surgeons in Pakistan: A Multicenter Cross-Sectional Study

**DOI:** 10.7759/cureus.26997

**Published:** 2022-07-18

**Authors:** Sana Zeeshan, Mehdia Rajab Ali, Rehan N Khan, Asad R Allana, Nida Zahid, Muhammad Kazim Najjad, Arslan A Abro, Muhammad Ali Nadeem, Zeeshan Mughal, Irshad Ahmed, Amjad Ali

**Affiliations:** 1 Breast Surgery, Aga Khan University Hospital, Karachi, PAK; 2 Pediatrics and Child Health, Aga Khan University Hospital, Karachi, PAK; 3 Urology, Al-Amiri Hospital, Kuwait City, KWT; 4 Orthopedics, Liaquat National Hospital and Medical College, Karachi, PAK; 5 Epidemiology and Public Health, Aga Khan University Hospital, Karachi, PAK; 6 Orthopedic Surgery, Liaquat National Hospital and Medical College, Karachi, PAK; 7 Surgery, Ziauddin University, Karachi, PAK; 8 Neurosurgery, Patel Hospital, Karachi, PAK; 9 Surgery, Jhalawan Medical College and Teaching Hospital, Khuzdar, PAK; 10 Breast Surgery, Hayatabad Medical Complex Peshawar, Peshawar, PAK

**Keywords:** surgeons, srq-20, psychological distress, depression, covid-19

## Abstract

Introduction

The severe acute respiratory syndrome coronavirus 2 (SARS-CoV-2) pandemic left a profound and pervasive impact on the healthcare infrastructure on a global scale. Since its onset, the pattern of reported cases and its associated mortality had shown variability with intermittent peaks causing a significant effect on the psychological well-being of the surgeons of Pakistan. The aim of this study was to assess the effects of the COVID-19 pandemic on the mental well-being of surgeons in Pakistan.

Methods

This multicenter cross-sectional study was carried out to assess the impact of COVID-19 on the psychological well-being of surgeons in Pakistan. The validated Self-Reporting Questionnaire-20 (SRQ-20) tool was circulated electronically via Google Forms (Google, Inc., Mountain View, CA, USA) in the practicing surgical fraternity across all five regions of Pakistan, i.e., Sindh, Punjab, Baluchistan, Khyber Pakhtunkhwa (KPK), and Azad Jammu and Kashmir (AJK).

Results

This study showed that the female gender, having fewer years of working experience, non-satisfaction with the available personal protective equipment (PPE), and working in the public sector were the factors affecting the psychological well-being of surgeons during the pandemic.

Conclusion

Considering the continuous rise in new cases during the ongoing pandemic, the mental health of surgeons working in low- and middle-income countries (LMIC) such as Pakistan has been significantly affected. There is an undeniable need to pay close attention to their psychological well-being. Measures need to be undertaken to ensure their physical and mental health and wellness.

## Introduction

Severe acute respiratory syndrome coronavirus 2 (SARS-CoV-2) was first identified in December 2019 in China and was subsequently declared a pandemic by the World Health Organization (WHO) on March 11, 2020, based on the alarming levels of its spread and severity [1]. The first case of COVID-19 in Pakistan was reported from Karachi on February 26, 2020 [2]. Since then, the number of confirmed cases in the country has been on a steep rise with 1,544,910 cases as of July 15, 2022 [3]. The rising numbers of the affected have had a profound and pervasive impact on the healthcare system, including the infrastructure and healthcare providers. Apart from creating a scarcity of resources and derailing the functional framework, it has changed every hospital’s dynamic, workflows, bedside manners, safety and surgical protocols, education, and training, to name a few.

While surgeons are usually not working on the frontlines in the face of the pandemic, with the increase in local spread, the surgical community was equally exposed to the contagion as other healthcare workers (HCWs). Moreover, with the shortage of healthcare staff, surgical personnel had started joining the frontline workforce [4]. Despite the postponement of elective surgical cases and reduction in outpatient clinics, surgeons continued to deal with emergencies such as acute trauma cases and in the surgical intensive care unit, exposing themselves to the virus [4-7]. Given the coronavirus’ mode of transmission, surgeons who specialize in the diseases of the aerodigestive tract, such as dental surgeons, otolaryngologists, and maxillofacial surgeons, and/or work in close proximity to it, such as ophthalmologists and general surgeons, are more susceptible than others due to the generation of aerosols from the procedures involved [8-11]. Surgeons are experiencing fatigue, the challenges of clinical redeployment, and skin damage due to prolonged use of personal protective equipment (PPE) [4,12]. When a surgeon requires isolation either due to suspicion or a definitive diagnosis of COVID-19, social isolation from family takes an emotional toll [4]. Surveys conducted in some countries to assess the impact of COVID-19 on surgeons showed that surgeons are concerned about their personal safety, their family’s safety, patient’s safety, their career and training, and financial setbacks, with the younger surgeons being more concerned than the experienced ones [4,13,14]. Working amidst an infectious outbreak poses the risk of developing “health anxiety” and “critical incident stress syndrome,” which can impair the ability to make decisions and can affect the surgeon’s private and social life [15,16]. With the closure of schools and child daycare facilities during the pandemic, surgeons with kids faced the added stress of finding caretakers to look after their children [17,18]. Most hospitals had suspended nonurgent, elective procedures to reallocate nurses, anesthetists, ventilators, surgical wards, and theaters for patients with COVID-19. Because of this, surgeons also dealt with their patient’s dissatisfaction with the indefinite postponement of their surgery.

While the protocols and principles for surgical procedures in times of COVID-19 exist and continue to grow, there is a paucity of quantitative documentation on how this pandemic has affected the mental health of surgeons in the short and long term. This study will help us understand the psychological impact of COVID-19 on the surgical fraternity of a low- and middle-income country (LMIC), such as Pakistan. The data obtained will also allow the comparison of surgeons’ psychological well-being, in terms of mental distress (depression and anxiety), belonging to the public and private sector hospitals of the country. This will be important considering that similar studies have not previously been conducted in the region. Additionally, a comparison of results with available data from both developed and developing parts of the world will help in identifying the severity of the problem among the surgeons of Pakistan in relation to published literature.

## Materials and methods

Objectives

The objectives of this study were to determine the psychological effects of the COVID-19 pandemic in terms of psychological distress, depression, and anxiety on practicing surgeons of Pakistan and its comparison between surgeons belonging to different subspecialties working in private and public sector hospitals and in the five regions of Pakistan.

Methods

This is a multicenter cross-sectional study in which an electronic survey of practicing surgeons in Pakistan was conducted to assess the impact of COVID-19 on their psychological well-being. Participant recruitment took place from five regions of Pakistan (Sindh, Punjab, Baluchistan, Khyber Pakhtunkhwa (KPK), and Azad Jammu and Kashmir (AJK)), and a proportionate sampling method was applied in each region.

Approval to conduct the study was taken from the Ethical Review Committee (ERC) of Aga Khan University Hospital, Karachi (ERC#2021-5802-15434). Informed consent (electronically) was obtained from the surgeons via Google Forms (Google, Inc., Mountain View, CA, USA), which was available online for two months.

The validated Self-Reporting Questionnaire-20 (SRQ-20) tool was used to measure psychological distress in surgeons. This 20-item self-reporting questionnaire is a screening tool developed by the World Health Organization (WHO) and is widely used in LMICs. The SRQ-20 has dichotomous response categories of “yes” and “no.” The total score for 20 items ranges from 0 to 20 [19].

The sample size was calculated using open epi software version 3.01. The minimum sample size of 1,613 surgeons was calculated from the number of registered surgeons in Pakistan as determined by Pakistan Medical Commission (PMC), with inflation of 10% for nonresponse rate, a precision of 3, and a design effect of 1.5. We were unable to determine the exact number of surgeons who were retired, practicing out of the country, or had passed away out of all the registered surgeons in PMC during sample calculation. Due to the low response rate, we were able to collect data from only 512 surgeons across Pakistan.

Surgeons from all subspecialties, i.e., cardiothoracic surgery, general surgery, neurosurgery, ophthalmology, and operative dentistry, and their subspecialties, including prosthodontics, orthodontics, and periodontology, oral and maxillofacial surgery, orthopedic surgery, otorhinolaryngology, pediatric surgery, plastic surgery, urology, breast surgery, surgical oncology, and vascular surgery, were included in the study. Data on social and demographic status, including age, gender, marital status, family structure, number of children, employment status and years of independent work experience, field of specialization, name of hospitals, city, and number of working hours per week were recorded, along with symptoms of depression and anxiety. Surgeons who were not in practice during the study period or who refused to consent to participate were excluded.

Statistical analysis

Data were exported from Google Forms to Microsoft Excel (Microsoft Corp., Redmond, WA, USA). Data were analyzed using Statistical Package for Social Sciences (SPSS) version 23.0 (IBM Corp., Armonk, NY, USA). For descriptive analysis, quantitative variables such as the number of children were reported as means ± SD/median (IQR) and were assessed using an independent t-test/Mann-Whitney U-Test (for comparison between private and public hospitals) and one-way ANOVA/Kruskal-Wallis test (for comparison between provinces). Qualitative variables such as gender, age, marital status, working experience, and anxiety were reported as frequencies and percentages and were assessed using the chi-square/Fisher’s exact test. Unadjusted and adjusted beta coefficient with their 95% confidence interval (CI) was reported using linear regression analysis to assess the factors associated with mental disorders of surgeons. Moreover, unadjusted and adjusted odds ratios (OR) with their 95% CI using ordinal regression analysis were reported to determine the factors associated with depression and anxiety among surgeons. The plausible interactions and confounders such as known cases of depression and anxiety and surgeon on antidepressants or anxiolytics recommended by a psychiatrist were also assessed. A p-value of <0.05 was considered significant throughout the study.

## Results

Table 1 shows the description of participants according to their sociodemographic status, which includes age, gender, marital status, and family structure; their work-related information, including region, type of hospital practice, years of work experience, and specialty; and their involvement with COVID-19, which includes caring for infected patients, availability of appropriate PPE, and if they or any of their close family members got infected by COVID-19.

**Table 1:** Sociodemographic status, work-related information, involvement with COVID-19, and mental health of surgeons as per the SRQ-20 sum score AJK: Azad Jammu and Kashmir, KPK: Khyber Pakhtunkhwa, PPE: personal protective equipment, IQR: interquartile range

	Total	Frequency	Percentage
		512	
Sociodemographic factors			
Age (years)	≤30	14	2.7%
	>30-40	260	50.8%
	>40-50	116	22.7%
	>50-60	92	18%
	>60-70	24	4.7%
	>70	6	1.2%
Gender	Male	365	71.3%
	Female	147	28.7%
Marital status	Single	60	11.7%
	Married	444	86.7%
	Divorced/separated	8	1.6%
Family structure	Nuclear family	214	41.8%
	Joint family	261	51%
	Living alone	32	6.3%
	Living with friends/acquaintances	5	1%
Work-related information			
Region of primary hospital	AJK	2	0.4%
	Baluchistan	38	7.4%
	KPK	24	4.7%
	Punjab	108	21.1%
	Sindh	340	66.4%
Private or government practice	Only government/municipal/public hospital practice	230	44.9%
	Only private hospital practice	282	55.1%
Years of work experience	<5 years	197	38.5%
	5-10 years	122	23.8%
	>10 years	193	37.7%
	Total	512	100%
Field of specialization	Breast surgery	32	6.3%
	Cardiothoracic surgery	10	2%
	Dental surgery or any of its subspecialties	36	7%
	General surgery	190	37.1%
	Neurosurgery	26	5.1%
	Ophthalmology	12	2.3%
	Orthopedic surgery	50	9.8%
	Otorhinolaryngology	26	5.1%
	Pediatric surgery	46	9%
	Plastic surgery	22	4.3%
	Surgical oncology	10	2%
	Urology	42	8.2%
	Vascular surgery	10	2%
COVID-19			
Involved in COVID-19 care	No	202	39.5%
	Yes	310	60.5%
Satisfied with the PPE availability	No	158	30.9%
	To some extent	128	25%
	Yes	226	44.1%
Infected by COVID-19	No	322	62.9%
	Yes	190	37.1%
Family members infected by COVID-19	No	208	40.6%
	Yes	304	59.4%
Mental health			
SRQ-20 sum	No symptoms present	129	25.2%
	Some symptoms of depression/anxiety	229	44.7%
	Considerable symptoms of depression/anxiety	154	30.1%
SRQ-20 median (IQR)		2 (5)	

A total of 512 surgeons responded to the online survey, of which 365 (71.3%) were males and 147 (28.7%) were females. Of these participants, 50.8% were in the age range of 30-40 years, and 86.7% were married. Of the respondents, 66.4% were from Sindh, 21.1% were from Punjab, and 7.4%, 4.7%, and 0.4% were from Baluchistan, KPK, and AJK, respectively. In addition, 44.9% primarily worked in government or public sector hospitals, whereas 55.1% were from private sectors. The majority of the participants (37.1%) were general surgeons. Also, 60.5% were directly involved in caring for COVID-19-infected patients, 44.1% were satisfied with the available PPE, 37.1% had been infected with COVID-19 at some point, and 59.4% had an infected close family member until the closure of data collection.

Table 1 also demonstrates the SRQ-20 sum score and median IQR, which includes three categories (no symptoms present, some symptoms of anxiety/depression present, and considerable symptoms of anxiety/depression present). Overall, 25.2% of the respondents had no reported symptoms, 44.7% had some symptoms, and around 30% had considerable symptoms of anxiety and depression.

Our study found that the odds of having considerable depression and anxiety symptoms during the COVID-19 pandemic were 2.061 times greater in female surgeons as compared to male surgeons (Table 2). The results also showed that in surgeons who had less than five years of working experience, the odds of having depression/anxiety symptoms during the pandemic were 1.747 times more compared to surgeons who had more than 10 years of working experience, while in surgeons having 5-10 years of working experience, the odds were 1.682 times greater compared to surgeons who had more than 10 years of working experience. In terms of PPE availability, our study also found that those surgeons who were satisfied with the provision of PPE showed a protective effect against depression/anxiety symptoms with the odds of 0.315 times compared to those who were not. Furthermore, our results found that surgeons who recovered from COVID-19 infections had 0.625 times the odds of developing depression/anxiety symptoms, hence showing a protective effect against such presentations.

**Table 2:** Adjusted OR for factors influencing SRQ-20 scores

	Covariates	Adjusted odds ratio	95% CI	p-value
Gender	Female	2.061	1.448,2.934	<0.001
Years of experience	<5 years	1.747	1.216,2.511	0.003
	5-10 years	1.682	1.122,2.521	0.012
Satisfaction regarding PPE availability	Yes	0.315	0.212,0.469	<0.001
	To some extent	0.435	0.286,0.662	<0.001
Had COVID-19 infection	Yes	0.625	0.452,0.863	0.004

Table 3 shows the relation between SRQ-20 score and the specialties of surgeons. Interestingly, the results of SRQ-20 data demonstrated a generally normal statistical distribution with the majority of the participants showing some symptoms of depression/anxiety (44.7%). The extremes of no or considerable symptoms are 25.2% and 30.1%, respectively. This table also shows that considerable symptoms of anxiety/depression were present in otorhinolaryngology specialists (42.3%), followed by neurosurgeons (38.5%), while some symptoms of depression/anxiety were present in ophthalmologists (83.3%), followed by neurosurgeons (61.5%). The specialty of cardiothoracic surgery showed up to 60% of surgeons with no symptoms.

**Table 3:** Specialty-wise distribution of SRQ-20 scores * % within the dominant field of specialization

Specialty	No symptoms present (number (%)*)	Some symptoms of depression/anxiety (number (%)*)	Considerable symptoms of depression/anxiety (number (%)*)	Total (number (%)*)
Breast surgery	8 (25%)	16 (50%)	8 (25%)	32 (100%)
Cardiothoracic surgery	6 (60%)	2 (20%)	2 (20%)	10 (100%)
Dental surgery or any of its subspecialties	14 (38.9%)	10 (27.8%)	12 (33.3%)	36 (100%)
General surgery	40 (21.1%)	85 (44.7%)	65 (34.2%)	190 (100%)
Neurosurgery	0 (0%)	16 (61.5%)	10 (38.5%)	26 (100%)
Ophthalmology	2 (16.7%)	10 (83.3%)	0 (0%)	12 (100%)
Orthopedic surgery	14 (28%)	22 (44%)	14 (28%)	50 (100%)
Otorhinolaryngology	8 (30.8%)	7 (26.9%)	11 (42.3%)	26 (100%)
Pediatric surgery	10 (21.7%)	20 (43.5%)	16 (34.8%)	46 (100%)
Plastic surgery	4 (18.2%)	10 (45.5%)	8 (36.4%)	22 (100%)
Surgical oncology	5 (50%)	5 (50%)	0 (0%)	10 (100%)
Urology	16 (38.1%)	20 (47.6%)	6 (14.3%)	42 (100%)
Vascular surgery	2 (20%)	6 (60%)	2 (20%)	10 (100%)
Total	129 (25.2%)	229 (44.7%)	154 (30.1%)	512 (100%)

On exploring the relation between SRQ-20 and marital status, there were considerable symptoms of anxiety/depression in surgeons who were unmarried (50%) and some symptoms of anxiety/depression in divorced surgeons (75%) during the COVID-19 pandemic. However, considering the limited number of divorced individuals (n=8), these results should not be considered conclusive.

Surgeons working in public sector hospitals had considerable symptoms of depression/anxiety during the COVID-19 pandemic in comparison to those working in private sector hospitals (40.9% versus 21.3%, p < 0.001) (Table 4).

**Table 4:** Comparison of SRQ-20 scores between public and private sector hospitals

Setting	No symptoms present (number (%))	Some symptoms of depression/anxiety (number (%))	Considerable symptoms of depression/anxiety (number (%))	p-value
Private	87 (30.9%)	135 (47.9%)	60 (21.3%)	<0.001
Public	42 (18.3%)	94 (40.9%)	94 (40.9%)	<0.001

Table 5 shows the relation between SRQ-10 scores and the province where the respondents were practicing. The p-value of 0.162 is insignificant, showing no relationship between depressive symptoms and the province during the COVID-19 pandemic.

**Table 5:** SRQ-20 sum across geographic locations

Province/region	No symptoms present (number (%))	Some symptoms of depression/anxiety (number (%))	Considerable symptoms of depression/anxiety (number (%))
Sindh	93 (27.4%)	148 (43.5%)	99 (29.1%)
Punjab	28 (25.9%)	51 (47.2%)	29 (26.9%)
KPK	2 (8.3%)	10 (41.7%)	12 (50%)
Baluchistan	6 (15.8%)	18 (47.4%)	14 (36.8%)
AJK	0 (0%)	2 (100%)	0 (0%)

## Discussion

The COVID-19 pandemic had a considerable impact on healthcare systems worldwide. This is the first large-scale nationwide survey in Pakistan to investigate the psychological effects of the COVID-19 pandemic on surgeons, to the best of our knowledge. The results showed that depression and anxiety were more common in female surgeons compared to male surgeons during the COVID-19 pandemic (OR: 2.061, 95% CI: 1.448,2.934). This result seems consistent with the findings of Albert, which described the incidence of developing major depression to be of higher occurrence in women than in men, with a 1.7-fold prevalence of global disability from the disorder in the former [20]. The author further describes that twice as many females develop depression than males, in the mid-teens to mid-20s (14-25 years), with an equal prevalence only after the age of 65 years, irrespective of cultural, occupational, or socioeconomic background [20]. Our results also showed that in surgeons having work experience of <5 years (OR: 1.747, 95% CI: 1.216,2.511) and having experience of 5-10 years (OR: 1.682, 95% CI: 1.122,2.521), the odds of having depressive symptoms were more compared to surgeons having work experience of more than 10 years. This result was inconsistent with previous literature. A study conducted by Scheepstra et al. showed an increase in the prevalence of depression in the groups with more years in practice (OR: 2.95, p=0.030, 95% CI: 1.11,7.80); however, it was consistent for anxiety symptoms as it showed a significant decrease in the prevalence of anxiety with more years in practice (OR: 0.33, 95% CI: 0.17,0.64) [21].

Our study showed that among the surgeons who were either satisfied completely or satisfied to a certain extent with the available PPE, the odds of having depression and anxiety symptoms were 0.315 and 0.435, respectively, compared to the surgeons who were not satisfied with the available PPE showing protective effect of the availability of PPE against depression and anxiety. This result was consistent with the previous literature as the study conducted by Lai et al. showed that 94% of the doctors who were not satisfied with the PPE were feeling unprotected and were more likely to have anxiety and depression [22]. Our study showed that surgeons who were infected with COVID-19 showed protective effect (OR: 0.625) against depression and anxiety symptoms; these results were inconsistent with the study conducted by Liu et al., which showed that during the COVID-19 pandemic, healthcare providers who experienced quarantine were more likely to report anxiety, irritability, depression, low concentration, lower work performance, and unwillingness to return to work [23].

The results showed that considerable symptoms of depression and anxiety (around 42.3%) were present in otorhinolaryngology specialists, which is consistent with the previous literature that stated that otolaryngologists and head and neck surgeons are at significant risk of exposure and are more likely to suffer from depression and anxiety due to COVID-19 [24]. Our study showed that around 40 (7.8%) surgeons had a prior clinical diagnosis of depression, which is a major factor in developing severe symptoms of depression and anxiety during the pandemic. Out of 40 surgeons, about eight surgeons sometimes experienced considerable symptoms of depression and/or anxiety, followed by six surgeons in whom symptoms occurred every two weeks during the pandemic. A study conducted by Civantos et al. supported this, which showed a self-reported history of a prior psychological condition as a significant risk factor for developing severe symptoms of depression and anxiety [25].

The results showed that surgeons working in public sectors had a considerable number of symptoms of depression and anxiety (40.9%) during the pandemic as compared to private sectors. This result was inconsistent with the previous literature. A study conducted by Amin et al. showed that there was no association between depression and anxiety and surgeons working in public or private sectors [26].

Our study is the first of its kind to explore, document, and quantify the psychological health of surgeons in Pakistan during the COVID-19 pandemic. It is a multicenter study targeting all regions of the country, and hence, the results reflect responses from all surgical specialties and surgeons working at both public and private sector hospitals. Furthermore, the factors associated with adverse mental health outcomes such as demographic factors and modifiable factors such as availability of PPE have been identified, which can guide direct policy changes to improve mental health outcomes.

A likely limitation of this study is, firstly, the low response rate. It is also possible that the surgeons who have been the most affected by this pandemic are the ones who are most likely to respond to this survey, thus leading to biased data collection.

Secondly, this study does not assess or record the trend of the impact of this pandemic. Since the data gathered represents the status of the population during a very small time, it falls short of representing how the impact may have changed based on the progression/containment of the pandemic in the months to follow.

Thirdly, this study does not attempt to distinguish between preexisting mental health symptoms and new-onset symptoms. Although we have attempted to identify some factors that may influence mental health, chiefly those relating to family structure, a more detailed account of such matters may be explored in future studies.

Lastly, there was a reduced response rate from surgeons of remote areas, particularly from two northern regions of Pakistan, i.e., KPK and AJK, which makes our results mostly generalizable for surgeons practicing in hospitals of Sindh and Punjab.

## Conclusions

With the continuous fluctuation in the prevalence of COVID-19, the mental health of surgeons in Pakistan has been in jeopardy. This study showed that the female gender, having fewer years of working experience, non-satisfaction with the available PPE, and working in the public sector are the major factors that affect the psychological well-being of surgeons during the pandemic. It is, therefore, necessary to pay close attention to their psychological well-being as well. Strategies such as the provision of adequate PPE, positive lifestyle behaviors, adequate rest between shifts, and psychotherapy sessions can be considered to ensure that the mental health status of surgeons does not affect their performance. Measures can also be implemented by the institutions, such as institution-based peer support programs, psychological online courses, providing a comfortable place for rest and destressing, and stress-relieving leisure activities. This circle of interventions for surgeons in the workplace can help toward safer and more productive results. Further research should be carried out periodically to assess the surgeons’ mental health at regular intervals and ensure their well-being during stressful circumstances.
